# The Impact of Diagnosis of Human Papillomavirus (HPV) Infection and Electrosurgical Excision Procedure (LEEP) for Cervical Intraepithelial Neoplasia 3 (CIN3) on Women’s Sexual Lives

**DOI:** 10.3390/diagnostics14090911

**Published:** 2024-04-27

**Authors:** Maria Teresa Bruno, Giuseppe Caruso, Elena Torrisi, Raffaela Grimaldi, Biagio Abate, Francesco Saverio Luciani, Susanna Basile, Marco Marzio Panella

**Affiliations:** 1 Department of General Surgery and Medical-Surgical Specialties, Gynecological Clinic, University of Catania, 95123 Catania, Italyelena-torrisi@hotmail.com (E.T.); raffaelaluisa.grimaldi@gmail.com (R.G.);; 2 Multidisciplinary Research Center in Papillomavirus Pathology, University of Catania, 95123 Catania, Italy; 3 Methods and Models Department for the Economy, Territory and Finance, La Sapienza University of Rome, 00185 Rome, Italy; francescosaverio.luciani@uniroma1.it; 4 Psychologist and Clinical Sexologist, 95123 Catania, Italy; susabasile@gmail.com

**Keywords:** cervical intraepithelial neoplasia, human papillomavirus, LEEP, quality of life, sexual health

## Abstract

The aim of the study was to assess sexual health in women who underwent Loop Electrosurgical Excisional Procedure (LEEP) for the treatment of cervical intraepithelial neoplasia 3 (CIN 3). One hundred thirty-one women were enrolled, and the Female Sexual Function Index (FSFI) questionnaire was administered before LEEP and 6 months after the procedure. In almost all of the participants, data revealed a statistically significant worsening in sexual quality of life after LEEP. Therefore, clinicians should be aware of these possible negative effects on sexual behavior, and provide women with appropriate, wide-ranging, and detailed counseling. The data obtained in the present study should help to plan appropriate counseling from communicating HPV diagnosis and medical treatment to CIN3 surgical procedure.

## 1. Introduction

The Papillomavirus (HPV) infection is the most common sexually transmitted disease in the world. The affinity of the virus for metaplastic squamous cells has allowed high-risk genotypes (HPV hr) to correlate closely with cervical cancer (SCC) [1,2]. HPV infection is responsible for almost all cervical cancers (~100%), the majority of anal (~88%) and vaginal cancers (~78%), and substantial proportions of penile (~51%), vulvar (≥25%), and oropharyngeal (30–70%) cancers. The high-risk genotypes 16 and 31, the most common in Europe [3], have a great oncogenic capacity; HPV 16 alone is responsible for more than 50% of cervical cancer cases worldwide. Studies conducted in recent decades have shown that papillomavirus infection can cause adverse events in pregnancy (spontaneous abortion, preeclampsia, and preterm birth) due to ability of HPV to infect and replicate in syncytiotrophoblast cells, causing the suffering and death of trophoblast cells [4,5,6].

The natural history is well known; the infection affects 80% of sexually active young women who have clearance of the virus within 12–18 months. Only in cases of persistence of the virus is there a high risk of progression and development of neoplastic lesions of the genital tract [7,8,9]. The transition from persistent infection to preneoplastic lesion and finally to invasive carcinoma appears to take decades in most cases with a minimum latency time of approximately 7 years. The development of high-grade precursors and cervical cancer appears to depend almost exclusively on infection of the totipotent reserve cells located in the squamocolumnar junction (SCJ) at the border between the ecto- and endocervix. The high susceptibility of these cells to HPV-induced oncogenic cell transformation explains why cervical cancer is much more common than primary vaginal tumors, although exposure to HPV is identical for the vagina and cervix.

The well-known determinants of progression are the viral genotype [8], the persistence of the infection, and the immune status of the woman, all factors that allow the integration of viral DNA into cellular DNA. Additional established factors of progression include prolonged use of oral contraceptives, tobacco smoking, and co-infection with other sexually transmitted agents such as Chlamydia trachomatis and Herpes Simplex Virus type 2 [10,11]. Penetrative vaginal or anal intercourse is not a necessary prerequisite for acquiring the infection of this virus, because it can be transmitted via direct contact with skin or mucosa, during intimate contacts of the genitalia or other mucosal surfaces infected.

Cervical cancer remains a primary cause of worldwide deaths in women, particularly in low-resource areas. Although HPV vaccines have proven effective, high-grade cervical intraepithelial neoplasia (CIN3), the precursor to cervical cancer, still represents a global health burden even in high-resource settings. The prevention of cervical cancer consists in the diagnosis and treatment of preneoplastic lesions.

Therefore, according to the American Society for Colposcopy and Cervical Pathology (ASCCP), all CIN3 lesions must be surgically treated [12,13]. Currently, the most common first-line technique is the Loop Electrosurgical Excisional Procedure (LEEP), which consists of the removal of the anomalous transformation zone in the shape of a cone (conization) through a diathermic loop. Historically, the treatment of choice for CIN3 was the cold knife cone (CKC), but its applicability has been limited by significant intraoperative and postoperative bleeding, perioperative infectious risk, and a recognized association with post-procedure cervical stenosis [14]. Subsequently, alternative excisional procedures such as laser conization and loop electrosurgical excisional procedure (LEEP) were developed. Laser conization has the advantage of being performed under local anesthesia with less associated bleeding and more accurate customization of cone size. However, thermal damage could make margin assessment difficult. LEEP has gradually replaced CKC and laser conization.

In general, it appears that excision of the uterine cervix carries a significant risk of preterm birth. The cervix plays an important role as a barrier against ascending infections during pregnancy. The cervical mucus plug that forms during pregnancy contains a series of antimicrobial substances, such as lysozyme, lactoferrin, and immunoglobulins, which play a key role in innate and adaptive immunity, a fundamental defense against pathogens. After an excision procedure, the cervix heals by regeneration of the ectocervical components and generation of scar tissue, but limited regeneration of the endocervical glands responsible for producing cervical mucus can lead to decreased immune function and are predisposed to tract infections upper with rupture of membranes and preterm birth [15]. An alternative theory for the association between excision procedures and preterm birth is a consequence of the removal of a substantial portion of cervical connective tissue, thus weakening the supportive capacity of the cervix as the pregnancy progresses [16].

CIN 3 typically affects young women often eager for offspring at the time of diagnosis, so the association between excision procedures and preterm birth has potentially significant reproductive and psychological consequences. Furthermore, there is a lack of research on the effects of this procedure on sexual function and behavior. It is important to highlight that the woman who undergoes LEEP already has a dysfunctional psycho-sexual behavior [17]. Usually, the woman is young, sexually active, and was diagnosed years earlier with papillomavirus infection, which is transmitted very easily during sexual intercourse with a partner who carries the virus. The diagnosis unleashes emotions such as surprise, bewilderment, anxiety, and fear, which are replaced by shame due to the prejudice that every sexually transmitted disease brings with it: being identified as promiscuous. The diagnostic–therapeutic process makes her feel sick and as if her femininity has been violated, which leads her to destroy her image and self-esteem, perceiving sexuality as a source of danger [18,19]. Trust in the couple vacillates, and doubt often creeps in towards the partner. Suspicion and distrust arise, even resulting in attacks of jealousy, especially in cases where the woman has temporarily stopped any type of intimate and sexual exchange. Procedures such as LEEP can be reduced thanks to the prevention of CIN through anti-HPV vaccines, whose diffusion in the population will reduce the circulation of the virus and consequently the negative psychological impact that an anomalous test and/or the presence of preneoplastic lesions have on women’s sexual life. Opinions regarding the impact of LEEP on women’s health are conflicting. Some studies found no change, while some studies highlighted the potential negative effects on fertility, pregnancy outcomes, and sexual health, increasing pre-existent psychosexual vulnerability [20,21]. Therefore, the aim of this study is to evaluate the specific effects of cervical conization with LEEP on the quality of sexual life of women with CIN3.

## 2. Materials and Methods

The observational study was performed at the Colposcopy and Minimally Invasive Surgery Service of the Gynecological Clinic, Department of General Surgery and Medical Surgical Specialties, University of Catania, Italy. The study protocol complies with the guidelines of the Declaration of Helsinki of 2013 and conforms to the Committee on Publication Ethics (COPE) guidelines. The study protocol was notified, in accordance with current legislation on observational studies provided by AIFA, to the Catania1 Ethics Committee of the University Hospital of Catania, which did not request additions or modifications to the protocol, in accordance with current legislation (20 March 2008). It was carried out from 2019 to 2021. Women who had been diagnosed with CIN3 and who would undergo LEEP were invited to participate in the study. Each woman was informed in advance about the objectives of the study and was asked to read and sign the study’s Informed Consent; none of them received any monetary compensation. Women with difficulty understanding written informed consent, or with psychiatric, neurological, and systemic diseases that could have affected sexual functions or with relationship difficulties, or having chronic pelvic and urinary system pain, pregnancy, or any other conditions that could potentially affect sexual activity, were excluded from the study. One hundred thirty-one women gave their consent to participate in the study.

### 2.1. Instrument

#### 2.1.1. Female Sexual Function Index (FSFI)

Sexual behavior was assessed using the self-administered female sexual function index (FSFI), validated in the Italian gynecological population of childbearing age [16]. The FSFI consists of 19 questions, classified into six domains; namely, desire, arousal, lubrication, orgasm, satisfaction, and dyspareunia, which are measured on a five-point Likert scale, ranging from 0 (no sexual activity) or 1 (never/very low) to 5 (always/very high). A score is calculated for each of the six domains and the total score is obtained by adding all the elements. The total score range is from 2 to 36. A value of ≤26.55 (=cut-off) is generally considered for the diagnosis of sexual dysfunction. The FSFI was administered before (T0) and 6 months after LEPP (T1).

#### 2.1.2. The Visual Analogic Scale (VAS)

The Visual Analogic Scale (VAS) was used to define genital pain [17]. Finally, at baseline, contraceptive counseling was offered to all women. Additionally, they were asked about the type of contraceptive they were using. Each woman was informed about the use of the condom as a useful contraceptive to protect herself from sexually transmitted infections. At follow-up, women were asked if they were continuing to use the same contraceptive or if they had switched methods after LEEP.

#### 2.1.3. Colposcopy

Colposcopy was performed using a Zeiss OPM1F colposcope (Carl Zeiss, Jena, Germany). We evaluated the visibility of the squamous columnar junction (SCJ) and studied the reactivity of the squamous epithelium to the application of acetic acid before and after Lugol’s solution. After. We used the nomenclature proposed by the International Federation for Colposcopy and Cervical Pathologies (IFCPC) in three grades of abnormality increasing according to severity: (i) Abnormal transformation zone (ATZ) grade 1 (ATZ1); (ii) grade 2 (ATZ2); or (iii) cancer. If lesions were evident, targeted biopsies were performed by portio.

#### 2.1.4. LEEP

LEEP was performed with colposcopic guidance under local anesthesia in the clinic by experienced personnel; loops that were 20 mm wide and 12, 15, or 20 mm deep were used depending on the characteristics of the lesion and the conformity of the cervix; in all cases, attention was paid to the customization of the dimensions of the loop used. The resection margins were kept 2–3 mm beyond the lesion, and the completeness of the lesion removal was verified colposcopically. A histological examination of the cervical cone established the definitive histological diagnosis and evaluated the involvement of the cone margins. The margins of the cone were reported as positive if the distance between CIN2+ and the resection surface was <1 mm.

### 2.2. Statistical Analysis

For comparisons of the values obtained from the FSFI items between the baseline and the follow-ups, the nonparametric Wilcoxon rank-sum test with z values was used. Furthermore, the differences between the values of each item obtained at T0 compared to those obtained at T1 were calculated. Consequently, the percentages of improvement, worsening, or no change were defined. Paired Student’s t-test was used to compare the values obtained at T0 with those of T1 from the VAS. Scores are presented as mean ± SD. The result was statistically significant when *p* < 0.05. Statistical analysis was carried out using the Primer of Biostatistics statistical computer package (Glantz SA, New York, NY, USA: McGraw-Hill, Inc. 1997).

## 3. Results

Table 1 shows the demographic characteristics of the women participating in the study. Mainly, it shows that the women in the sample had more than one partner and, in particular, 43.5% had more than three partners. In addition, the majority of the women (88.5%) were not using any barrier contraceptive, such as condoms. These findings could correlate with an increased risk of HPV infection.

At T1, 14 (10.7%) women dropped out of the study. Therefore, 117 (89.3%) women completed the study. Table 2 shows the statistical comparisons of the FSFI scores for each sexual item observed at T1 with respect to the T0 values. Each item had a reduction in value. Moreover, the T1 FSFI total score was less than that at T0 (*p* < 0.001). However, the total FSFI score was already dysfunctional at T0 as it was below the cut-off.

**Figure 1:**
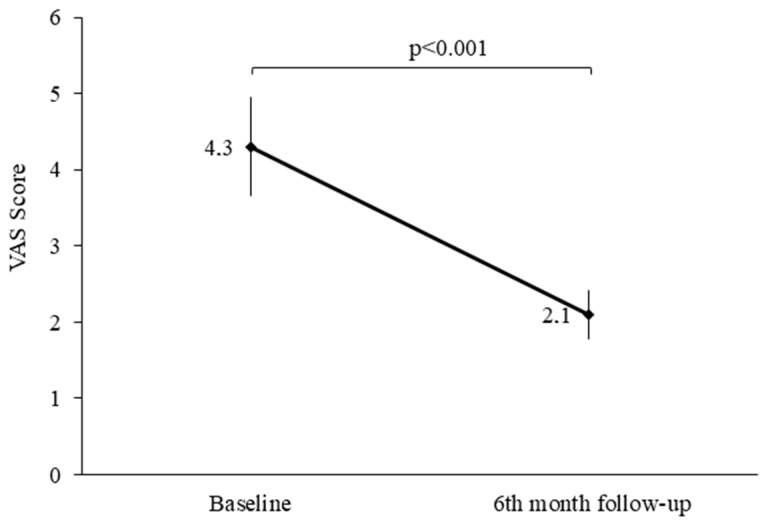
Visual Analog Scale (VAS) score of women with diagnoses of CIN3, before and after LEEP.

Figure 1 shows the variation in the VAS score, before and after LEEP.

A worsening of the score was observed at T1 compared to the T0 values (*p* < 0.001).

Table 3 shows the qualitative and quantitative aspects of sexual function and sexual experience of women after LEEP. Mainly, women reported an overall worsening of sexuality. Specifically, the most evident data was increased sexual pain. In fact, 69.5% of women complained of increased dyspareunia, which could have negatively influenced all the other investigated items: lower orgasmic satisfaction and decreased sexual desire, arousal and lubrication.

A lower proportion of women reported improvement (from 4.6% to 13%), or unchanged sexuality (from 23.7% to 38.1%). Fear of having sexual intercourse increased in 62.6% of women; it was unchanged or decreased in 29.8% and 7.6% of women, respectively. All this affects intimacy; in fact, 64.9% of women reported a worsening after the procedure. On the other hand, 26.7% and 8.4% of women reported no change or better intimacy after the procedure, respectively. Moreover, before the procedure, 60.6% of women had a permanent partner, while 39.4% had an occasional partner. After the procedure, 36.6% had the same partner, 46.6% had a different partner, and 16.8% had no partner.

As mentioned above, at T0 88.5% of women did not use any contraceptive. At T1, the percentage decreased to 52%, which is −41.27%. On the other hand, women on hormonal contraceptives improved from T0 (3.1%) to T1 (6.2%) of 100%. Interestingly, the number of women who were using condoms increased from 8.4% to 42.7%. This improvement was 408%.

Finally, Table 4 shows the main studies that investigated the effects on sexual function of undergoing LEEP.

## 4. Discussion

The study aimed to explore the side effects following LEEP in women previously diagnosed with CIN3. The main evidence observed from the study results was a worsening of both pain symptoms and sexual function in women after the procedure.

The cervix is a key organ in a woman’s sexuality for structural, functional, and symbolic reasons. Many researchers have focused on the physiological role of the cervix in sexual function and have argued that the cervix may play a distinct role in sexual function during vaginal intercourse because it is rich in blood vessels, innervated by the uterosacral plexus, and forms the vault of the vagina. In a similar context, several studies have investigated whether total removal of the cervix during hysterectomy alters female sexual function compared to preservation of the cervix. However, the results remain controversial [22]. LEEP can compromise the vascular supply and innervation of the cervix, causing pain during intercourse or negatively influencing the woman’s sexual function.

Nevertheless, it should be noted that surgical removal of a portion of the cervix using LEEP could exacerbate the already compromised psycho-sexual health caused by HPV positivity. In fact, before receiving a CIN3 diagnosis, the woman often has a long history of HPV infection, affecting her sexual well-being. Indeed, various studies have demonstrated a negative impact of HPV infection diagnosis on psychological and sexual function [23,24,25,26]. The woman who has contracted HPV may feel ashamed, less feminine, fearful, and anxious; lastly, the relationship with her partner may go into crisis [27].

The present study showed that the sexual function of the women measured using the FSFI, at T0, had a total score of 19.8 ± 1.5; lower than the cutoff (<26.55) and therefore accepted for diagnosis of sexual dysfunction. The total score worsened after LEEP (15.7 ± 1.2, *p* < 0.001). All items of the FSFI contributed to the total score reduction; mainly dyspareunia, desire, and sexual satisfaction, but also arousal lubrication and orgasm experience. Indeed, as reported by various studies, this procedure could cause deleterious effects on women’s quality of life and sexual function, especially orgasm pleasure [28,29,30,31]. Litman et al. confirmed these results through a detailed and comprehensive review, which documented adverse effects on multiple domains, including lubrication, sexual pain, and desire, following LEEP [32]. However, other authors reported minimal or absent adverse effects on sexual function or improved quality of sexual life after LEEP [33]. Rahman MM et al. [34], in a cross-sectional study on the impact of LEEP on female sexual function, reported no changes in the frequency of sexual intercourse after LEEP. Inna N et al., using a self-designed questionnaire, and Serati M et al., the first to use a validated questionnaire (FSFI), concluded that, compared to sexual function before surgery, LEEP does not affect women’s sexuality [35,36]. Both authors attribute this result primarily to anxiety [18,19]. According to the authors, improvement might suggest that the psychological impact of HPV infection on sexual function could be greater than the anatomical and functional impact of LEEP. To support this, a consensus statement from the Fourth International Consultation on Sexual Medicine 2015 has shown that perceived poor health, mood, and anxiety disorders can cause dyspareunia, lack of desire, orgasm, and arousal [37]. They used a self-made questionnaire, which was not validated, and they perform the questionnaire only after 6 months after the procedure. In a systematic review about the adverse psychological outcomes following colposcopy and related procedures, 23 papers were assessed, and they found that a wide spectrum of anxiety, distress-related sexual function problems, and fears about future fertility to depression can occur after those procedures [38,39]. According to the authors, improvement might suggest that the psychological impact of HPV infection on sexual function could be greater than the anatomical and functional impact of LEEP.

**Table 4:** Major studies on sexual health in women undergoing LEEP for CIN.

Author/Year	Study Design	Study Population	Diagnosis	Age (Mean)	Follow-Up Time (Mean)	Tool Implemented	Significant Findings
Litman et al., 2022 [32]	review	Nine study	CIN2-CIN3				Adverse effects on lubrication, sexual pain, and desire following LEEP.
Heinzler, J et al., 2018 [33]	Case-control study		CIN	19–56	6 months	FSFI	Had the lowest sexual functioning
Rahman MM et al., 2017 [34]	cross-sectional study	46	CIN	25–40	6 months after LEEP	Self-designed Questionnaire	LEEP doesn’t significantly affect women’s sexuality,
Inna et al., 2010, Thailand [35]	Cross-sectional	89	CIN 1-3	24–57 (42)	12.1–70.9 weeks (29.3)	Self-designed Questionnaire	At up to 1 year follow-up: decrease in overall sexual satisfaction, orgasmic satisfaction, and vaginal elasticity
Serati et al., 2010 Italy [36]	Cross-sectional	58	CIN 1 persistent and CIN 2/3	22–39 (36)	At time of LEEP, and 6 months	FSFI	At 6 months follow-up: decrease in desire
Sadoun C et al., 2016 [37]	prospective	69	CIN 2-3	37.5 ± 7.9	three months after LEEP	auto-questionnaire BISF-W (Brief Index of Sexual Functioning for Women).	Sexual life assessed by the BISF-W is not altered post-operatively.
Hellsten et al., 2008 Sweden [38]	Cross-sectional	45	CIN 1 above age 30 y and CIN 2/3 at any age	23–49 (27)	At time of LEEP, 6 months, and 2 years	Psychosexual Questionnaire designed by Howells et al. [39]; STAI	At 2 years follow-up: decrease in spontaneous interest and frequency of intercourse

In the present study, 9.4% of women had an improvement in sexual life after the procedure; however, an average of 60.7% of women complained of a worsening sexual experience, while in 29.9% of the women, their sexuality was unchanged compared to before the procedure. Fear of having intercourse and/or intimacy with a partner worsened. This may have resulted in a change in the relational schema. In fact, after the procedure, 45.5% of women terminated their previous relationship, 16.8% had no partner, and 37.7% had the same partner. After the procedure, the type of contraception adopted by women also changed. In fact, the percentage of women who did not use any contraceptives decreased from 88.5% to 52% (−41.2%), mainly switching to condom use (from 8.4% to 42.7%), and modestly to hormonal contraception (from 3.1% to 6.2%). Previously, other authors reported similar results [38,39,40,41].

Repeated testing and painful and invasive treatments could also contribute to a sense of sexual dysfunction vulnerability. However, the specific effects of LEEP on the quality of sexual life are still debated and studies in the literature are lacking. It should be considered that the state of discomfort towards sexual life and depression experienced by women could depend on a lack of counseling or patient-centered care model by the provider before undergoing LEEP [31]. The provider should focus the information on the symptoms and sexual discomforts that could arise after the procedure, and intervene adequately on their appearance, either directly or via a multidisciplinary team [42].

The integrity of the cervix, previously defined as an erotogenic organ, is essential for healthy sexuality, and LEEP could lead to a drastic change in sexual experiences. As a precondition, it is important to emphasize the worldwide-accepted dense sensorial innervation of the cervix, confirming the paramount role of cervical stimulation on female sexual function [29,43,44,45,46].

Currently, LEEP is the gold standard in the treatment of CIN3. Given the high impact of LEEP on sexual life, in selected women who prefer non-invasive treatment, Imiquimod can be used as an alternative treatment. It is an immunomodulator, toll-like receptor agonist, capable of determining a local immune response at the level of the cervix through the secretion of proinflammatory cytokines. It has been approved for the treatment of various skin conditions and is recommended as a first-line treatment for vulvar HSIL. However, its efficacy and safety in the management of vaginal and cervical cancers are still being studied [47].

This study has strengths in assessing female sexual dysfunction and sexual distress using validated instruments, FSFI, and the Visual Analogic Scale (VAS) to define genital pain. A disadvantage is that studying sexual functioning with a questionnaire may be difficult. On the other hand, it has been observed that sensitive information is easier to disclose in a questionnaire than in an interview. A limitation of the study is the small sample size that reduces the power of interpretation, but the more important limitation of this study was the lack of pre-LEEP sex counseling. This may be the aim of a future case-control study.

## 5. Conclusions

The data obtained by the present study should help to plan appropriate counseling from communicating HPV diagnosis and medical treatment to CIN3 surgical procedure. In fact, HPV-infected women have a long history of hosting the virus, having unprotected sex, having had several partners, or having simultaneous sex with different partners. The physician should, from the time of the first diagnosis, address an educational approach to systemic and sexual health. Counselling fundamentally based on the correct use of condoms and the avoidance of promiscuous relationships is the most important approach in order to enable women to improve their quality of life, and their sex lives.

## Figures and Tables

**Table 1:** Demographic characteristics.

	Study Group (*n* = 131)
Mean Age (±SD)	±
Age (y)-*n* (%)	
25–30	59 (45.1)
>30–40	72 (54.9)
Age at the first intercourse	14.3 ± 2.1
Parity, *n* (%)	
None	50 (38.2)
One child	32 (24.4)
Two or more children	49 (37.4)
Education, *n* (%)	
None	27 (20.6)
Up to high school	67 (51.1)
College	37 (28.3)
Work, *n* (%)	
Teacher	24 (18.3)
Housewife	20 (15.3)
Trader	18 (13,7)
Self-employed	28 (21.4)
Employee	24 (18.3)
Student	17 (13)
Number of partners, *n* (%)	
One	12 (9.2)
Two	32 (24.4)
Three	30 (22.9)
More than three	57 (43.5)
Contraception, *n* (%)	
None	116 (88.5)
Condom	11 (8.4)
Hormonal	4 (3.1)
Smoking habit, *n* (%)	
Smoker	46 (35.1)
Never smoked	85 (64.9)
Alcohol usage, *n* (%)	
Yes	70 (53.4)
No	61 (46.6)

**Table 2:** Female Sexual Function Index (FSFI) scores at baseline (T0) and at the 6-month follow-up after LEEP (T1).

FSFI Items	T0	T1	p *
Desire	3.1 ± 1.4	2.1 ± 1.2	<0.001
Arousal	3.3 ± 1.5	2.9 ± 1.2	0.02
Lubrication	3.6 ± 1.9	3.2 ± 1.1	0.04
Orgasm	3.2 ± 1.9	2.7 ± 1.3	0.01
Satisfaction	3.2 ± 1.7	2.1 ± 1.4	<0.001
Dyspareunia	3.4 ± 1.9	2.7 ± 1.2	<0.001
FSFI Total score	19.8 ± 1.5	15.7 ± 1.2	<0.001

Values are means ± SD. * *p* values determined by non-parametric Wilcoxon’ s rank-sum test.

**Table 3:** Qualitative and quantitative aspects of sexual function and experience of women with diagnoses of CIN3 after LEEP procedure.

Item	Worse *n* (%)	Unchanged *n* (%)	Better *n* (%)
Desire	81 (61.8)	36 (27.5)	14 (10.7)
Arousal	69 (52.7)	50 (38.1)	12 (9.2)
Lubrication	68 (51.9)	49 (37.4)	14 (10.7)
Orgasm	79 (60.3)	35 (26.7)	17 (13)
Satisfaction	89 (67.9)	31 (23.7)	11 (8.4)
Dyspareunia	91 (69.5)	34 (25.9)	6 (4.6)
Fear of sexual intercourse	82 (62.6)	39 (29.8)	10 (7.6)
Intimacy with the partner	85 (64.9)	35 (26.7)	11(8.4)

## Data Availability

The datasets used and/or analyzed during the current study are available from the corresponding author upon reasonable request.
